# From task-specific AI to multimodal foundation models in PET/CT: a readiness framework for clinical translation

**DOI:** 10.3389/fnume.2026.1956567

**Published:** 2026-10-08

**Authors:** Amgad Muneer, Maliazurina B. Saad, Hui Xu, Jia Wu

**Affiliations:** 1 Department of Imaging Physics, The University of Texas MD Anderson Cancer Center, Houston, TX, United States; 2 Department of Thoracic/Head and Neck Medical Oncology, The University of Texas MD Anderson Cancer Center, Houston, TX, United States; 3 Institute for Data Science in Oncology, The University of Texas MD Anderson Cancer Center, Houston, TX, United States

**Keywords:** clinical translation, foundation models, molecular imaging, multimodal artificial intelligence, nuclear medicine, PET/CT, positron emission tomography, vision-language models

## Abstract

Artificial intelligence in PET/CT is evolving from narrowly optimized systems toward reusable pretrained representations and multimodal models. PET/CT is a demanding test case because clinically meaningful interpretation must preserve quantitative molecular information while integrating anatomic CT, acquisition metadata, longitudinal examinations, and free-text imaging reports. This focused narrative review synthesizes literature reported from January 2021 through July 2026, spanning PET reconstruction and enhancement, attenuation correction, segmentation, outcome modeling, pretrained representations, vision-language systems, and agentic workflows. We distinguish task-specific models, reusable pretrained representations, promptable models, vision-language systems, generalist foundation-model candidates, and agentic systems according to demonstrated capabilities, while separately considering evaluation benchmarks. Task-specific deep learning has demonstrated promising technical performance in low-count imaging and whole-body tumor analysis, but most evidence remains retrospective and restricted by radiotracer, disease, scanner, institution, or endpoint. Emerging PET-native studies demonstrate label-efficient segmentation, image-text grounding, report generation, and modular tool orchestration; however, they do not yet establish broad cross-tracer generalization or prospective clinical benefit. We therefore propose MOSAIC-PET, an author-developed heuristic six-domain evaluation framework for PET/CT foundation models encompassing Molecular fidelity, Organ and anatomic grounding, Semantic faithfulness, Adaptability, Integration and clinical impact, and Continuous governance. MOSAIC-PET has not undergone Delphi consensus or formal validation and is intended to organize PET-specific evidence requirements rather than function as a validated clinical-readiness tool. The near-term translational priority is the development of modular, auditable systems in which validated quantitative tools and evidence-linked language outputs remain under specialist oversight, with progression from external testing to silent evaluation, assisted-use studies, clinical-impact assessment, and continuous lifecycle monitoring.

## 1 Introduction

Artificial intelligence (AI) has entered nearly every stage of the PET imaging chain, including detector and acquisition optimization, image reconstruction, denoising, attenuation correction, lesion detection, segmentation, quantitative biomarker extraction, response assessment and outcome prediction. During the first wave of adoption, each model was generally optimized for one task and one narrowly defined data distribution. These systems can be highly effective, but their value often diminishes when tracer biology, scanner hardware, reconstruction settings, patient population or clinical question changes. Recent reviews of PET AI consistently identify external validation, standardization, and workflow integration as persistent bottlenecks (1–4).

Foundation models propose a different development strategy. Instead of learning each endpoint from scratch, a large model is pretrained on broad data using self-supervised, contrastive or generative objectives and subsequently adapted through prompting, lightweight fine-tuning, retrieval or task-specific heads (5). Medical vision-language models extend this strategy by aligning images with reports, electronic health records or other language supervision, thereby enabling zero-shot classification, cross-modal retrieval, visual question answering, and report generation. Large generalist biomedical models and three-dimensional CT vision-language models have demonstrated that diverse supervision can support multiple downstream tasks from a shared representation (6–9).

Nuclear medicine should not treat this transition as a simple transfer of methods from radiography or CT. PET is a quantitative, tracer-dependent measurement of biology. Uptake depends on injected activity, uptake time, reconstruction, motion, attenuation and scatter correction, partial-volume effects and the physiology of the radiopharmaceutical (1–3, 10). A model can produce a visually plausible image or fluent report while corrupting standardized uptake values (SUVs), suppressing small lesions, confusing physiologic and pathologic uptake, or inventing comparative findings. The central challenge is therefore to build models that align molecular signal, anatomy and clinical semantics without erasing the physical meaning of PET (11–13).

This focused narrative review has three aims. First, it maps the evolution from task-specific PET/CT AI to reusable and multimodal foundation models. Second, it critically evaluates emerging PET-native promptable and vision-language systems against the requirements of clinical nuclear medicine. Third, it proposes MOSAIC-PET, an author-developed conceptual framework for PET/CT foundation-model development, evaluation, and lifecycle governance. Existing PET-focused AI reviews have primarily examined task-specific applications and recurring challenges such as standardization, external validation, and workflow integration (1–4), whereas recent reviews of foundation models and vision-language systems have largely considered these developments across medical imaging more broadly (14–17). The distinctive contribution of the present review is to connect these two areas by examining foundation-model development through the quantitative, tracer-dependent, multimodal, and longitudinal requirements of molecular imaging. In addition, we provide an operational taxonomy that distinguishes emerging systems according to demonstrated capabilities and link these distinctions to PET/CT-specific translational evidence requirements. Because PET-native foundation, vision-language, and agentic systems have appeared in rapid succession during 2025–2026, a synthesis now is timely and may help establish an evaluation discipline before premature or overstated clinical claims become entrenched. The framework is intended to make the review actionable for nuclear medicine physicians, imaging scientists, AI developers, clinical trialists, and regulators. As illustrated in Figure 1, PET/CT AI evolves from narrowly optimized models toward reusable representations and multimodal foundation models capable of supporting segmentation, retrieval, quantitative analysis, reporting, and longitudinal reasoning.

**Figure 1:**
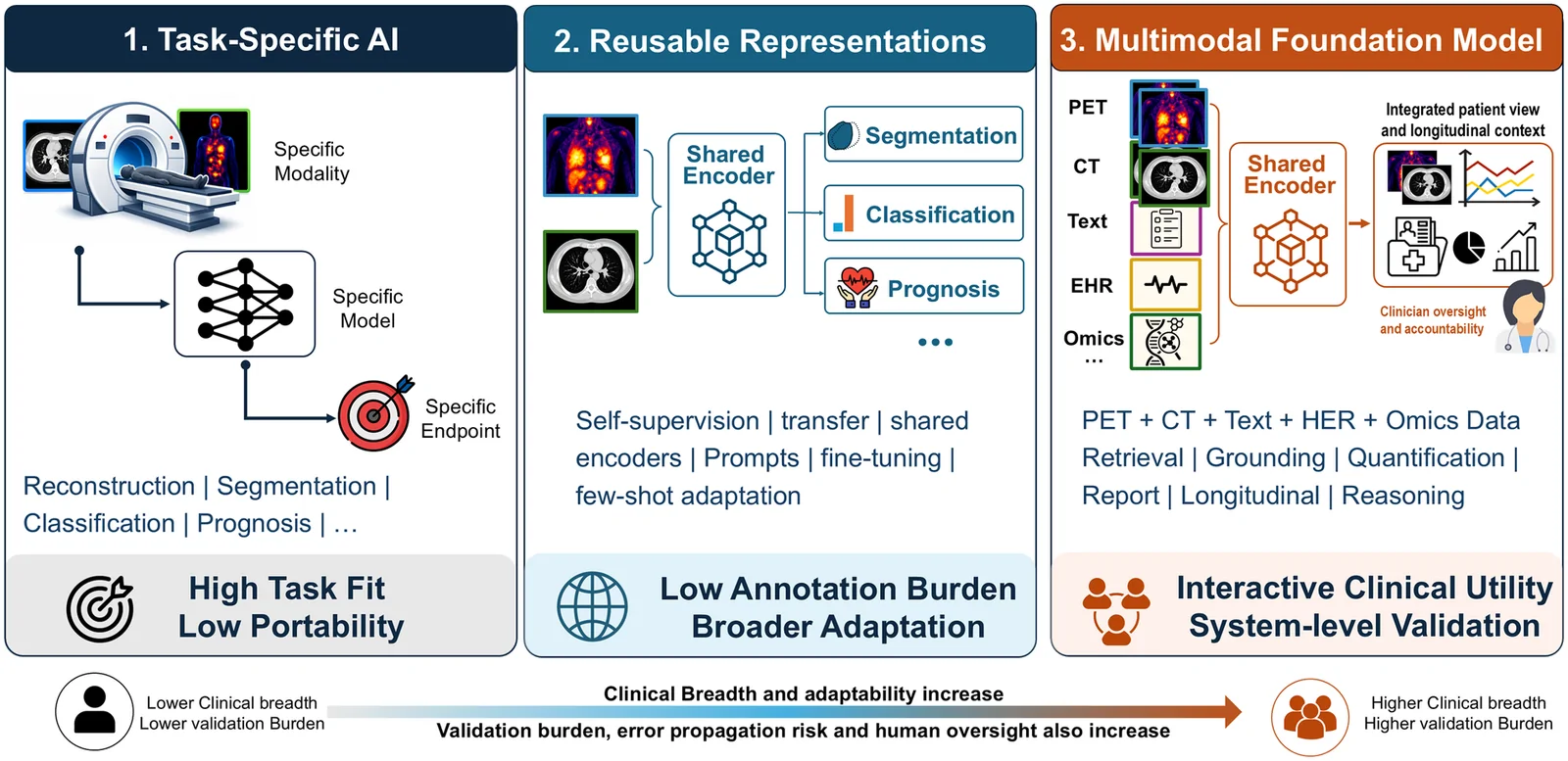
Evolution from task-specific PET/CT automation to multimodal foundation models. The transition expands adaptability and interaction but also increases the burden of validation because errors can propagate across tasks and outputs.

Accordingly, this review addresses three research questions: (1) How has PET/CT artificial intelligence evolved from task-specific models toward reusable pretrained representations, promptable systems, multimodal and vision-language models, and agentic workflows, and what capabilities have been demonstrated to date? (2) What PET/CT-specific technical, quantitative, biologic, and clinical requirements constrain the generalizability and translation of these emerging systems? (3) What evidence is needed to evaluate the readiness of PET/CT foundation-model systems for clinical translation, and how can these requirements be organized within a PET-specific evaluation framework?

### 1.1 Review scope, literature approach, and eligibility criteria

To address these research questions, we conducted a focused narrative search of PubMed, arXiv, and official conference proceedings repositories for English-language literature published from January 2021 through July 11, 2026. Search concepts combined nuclear medicine, positron emission tomography, PET/CT, total-body PET, artificial intelligence, deep learning, self-supervised learning, foundation model, multimodal model, vision-language model, segmentation, reconstruction, report generation, and clinical translation. The January 2021 boundary was chosen to focus the synthesis on the contemporary foundation-model era, while earlier standards and foundational studies were retained when necessary for technical, methodological, or historical context.

Eligible sources included peer-reviewed original studies, multicenter evaluations, conference proceedings, benchmark and challenge reports, public-dataset studies, relevant review or consensus articles, regulatory and reporting guidance, and recent preprints when directly relevant to emerging PET/CT foundation-model development. Studies were included when they addressed PET or PET/CT artificial intelligence, pretrained or reusable representations, foundation models, multimodal or vision-language learning, reconstruction or enhancement, segmentation, prediction, report generation, agentic workflows, or clinical translation. General medical-imaging foundation-model studies were included selectively when they provided directly relevant methodological or translational context for PET/CT. Sources were excluded if they were non-English, fell outside the January 2021–July 11, 2026 study period without a necessary foundational or standards-related role, lacked direct relevance to PET or PET/CT or directly applicable medical-imaging foundation-model methodology, or fell outside the AI and translational scope of this review. Because PET/CT foundation-model research is evolving rapidly, several of the most recent and directly relevant developments available at the time of the search were still available only as preprints. These reports were explicitly identified as non-peer-reviewed and interpreted as preliminary; peer-reviewed evidence and consensus or regulatory guidance were given greater weight in clinical and translational conclusions, and preprints were not used alone to support claims of clinical readiness.

## 2 Why PET/CT requires a domain-specific foundation-model strategy

### 2.1 PET is a molecular measurement, not only an image

PET intensities have quantitative and biologic interpretations that are more constrained than ordinary natural-image pixels. SUV-based response criteria, metabolic tumor volume, total lesion glycolysis and kinetic parameters can influence staging, treatment response assessment and prognosis. Accordingly, AI enhancement must be evaluated not only for image similarity but also for lesion detectability, uptake bias, repeatability and clinically meaningful thresholds. Multicenter low-count studies have shown that deep-learning enhancement can preserve diagnostic confidence and SUV agreement, while also underscoring the need for reader assessment and quantitative agreement appropriate to the intended use (10, 11, 18).

The same principle applies to generative and diffusion models. A denoiser may improve signal-to-noise ratio while changing lesion contrast, smoothing intratumoral heterogeneity or hallucinating uptake. Recent three-dimensional diffusion work has improved robustness across scanners, tracers and dose levels, yet clinical adoption still requires lesion-level error analysis and prospective comparison with the acquired reference data (19–21).

### 2.2 Hybrid imaging creates paired but asymmetric modalities

PET and CT are spatially linked but semantically asymmetric. CT provides high-resolution anatomy and attenuation information, whereas PET reveals tracer-specific physiology at lower spatial resolution. Naive channel concatenation, late fusion or independent encoders may underuse this complementarity or permit one modality to dominate. Cross-modal PET/CT models have improved prediction over single-modality approaches in specific clinical settings, including occult nodal metastasis in lung cancer, while self-supervised joint PET/CT denoising demonstrates the value of paired learning without fully aligned normal-dose targets (22, 23).

Hybrid acquisition also introduces failure modes that are not present in single-modality models: respiratory or patient motion can misregister uptake and anatomy; metallic implants and contrast can alter CT appearance; PET field of view and CT protocol may differ; and the CT component may be low dose or non-diagnostic (24, 25). PET/CT foundation models must therefore learn correspondence while retaining the ability to detect and communicate misalignment.

### 2.3 Tracer diversity is both an opportunity and a source of domain shift

FDG dominates existing AI datasets, but nuclear medicine spans PSMA-targeted agents, somatostatin-receptor tracers, amyloid and tau agents, amino-acid tracers, cardiac perfusion tracers and an expanding theranostic ecosystem. A representation that generalizes across tracers could enable label-efficient learning and rare-indication transfer. Conversely, anatomic uptake that is abnormal for one tracer can be expected for another. Reviews of PSMA PET/CT and total-body PET emphasize that tracer-specific biology and acquisition protocols cannot be treated as nuisance variation (26–28). Table 1 summarizes the principal differences between task-specific PET/CT AI and multimodal foundation models in terms of training objectives, supported modalities, adaptation strategies, outputs, and translational risks.

**Table 1:** Conceptual differences between task-specific AI and multimodal PET/CT foundation models.

Dimension	Task-specific AI	PET/CT foundation model	Translation implication
Training objective	One labeled endpoint	Broad self-supervised, contrastive or generative pretraining plus adaptation	Benefit must be demonstrated across multiple tasks, not inferred from scale
Modalities	Usually PET, CT or fixed fusion	PET + CT + reports + clinical and longitudinal context	Missing-modality behavior and provenance become safety requirements
Adaptation	Full retraining	Prompting, linear probing, adapters or fine-tuning	Low-shot gains require leakage-free and external testing
Outputs	Class, mask, image or risk score	Masks, retrieval, text, quantification and interactive answers	Evaluation must cover each output and cross-output consistency
Primary risk	Overfitting to task/site	Broad error propagation, hallucination and hidden domain bias	Human oversight and post-deployment monitoring are essential

## 3 From task-specific AI to foundation models in PET/CT

### 3.1 Reconstruction, denoising and attenuation correction

Reconstruction is one of the most mature PET AI applications. Deep networks can learn image-space denoising, direct or unrolled reconstruction, attenuation and scatter correction, and mappings between low-count and standard-count acquisitions. Recent works show a shift from convolutional post-processing toward physics-informed, self-supervised, diffusion and prompt-guided methods (2, 12, 29, 30). Foundation-model development is also extending to raw PET measurements. ALL-PET introduced a latent-diffusion model operating directly on projection-domain data (sinograms) and demonstrated reuse across low-dose reconstruction, attenuation correction, delayed-frame prediction, and tracer separation. Although the study illustrates reusable pretraining across PET image-formation tasks, it remains a preprint and requires independent multicenter clinical validation (31).

Potential clinical benefits include lower injected activity, shorter acquisition, improved throughput, and potentially more tolerable scanning. However, traditional perceptual metrics such as peak signal-to-noise ratio or structural similarity are inadequate if SUV bias, lesion conspicuity or response classification changes. Prospective, multivendor reader evidence remains much less common than retrospective technical validation (11, 18). Synthetic CT and CT-free attenuation correction add another layer of risk because errors can propagate into PET quantification. Recent studies of synthetic CT in PET and radiotherapy emphasize that evaluation must include attenuation maps, reconstructed PET bias and clinically relevant regional analyses, not only synthetic-image appearance (24, 25).

### 3.2 Detection, segmentation and whole-body tumor burden

Whole-body lesion segmentation is the bridge between task-specific models and foundation models. Public datasets and challenges have accelerated progress. The FDG-PET/CT-Lesions collection provided 1,014 studies with expert tumor annotations, while HECKTOR established reproducible PET/CT benchmarks for head-and-neck primary tumor and nodal segmentation and outcome prediction (32–34).

The autoPET challenge showed that accurate whole-body lesion segmentation is feasible and highlighted the importance of data quality and quantity alongside architectural design. Yet false-positive physiologic uptake, small lesions, diffuse marrow or splenic disease, post-treatment inflammation and uncommon cancers remain difficult. Challenge performance should therefore be interpreted as a benchmark under defined conditions, not evidence of universal clinical readiness (35, 36). Broader task-specific systems have begun to span diseases and tracers. A semi-supervised transfer-learning model evaluated 611 FDG PET/CT and 408 PSMA PET/CT scans, performed segmentation across several malignancies and derived prognostic measures for multiple downstream endpoints. This is an important step toward reusable representations, but the architecture still optimizes a defined family of tasks and depends on curated cohorts (37).

Federated learning offers one route to multi-institutional model development without centralizing images. In a six-center head-and-neck PET/CT study, federated transformer models approached centralized segmentation performance. Such approaches reduce data movement but do not automatically solve harmonization, annotation inconsistency, privacy leakage, governance or performance disparities across sites (38).

### 3.3 Diagnosis, response assessment and prognosis

Radiomics and deep learning have been applied to tumor characterization, nodal staging, therapy response and survival. More rigorous studies combine imaging with clinical covariates, use independent or prospective cohorts and evaluate added value beyond established comparators. For example, a PET/CT cross-modal signature for occult nodal metastasis in clinical N0 non-small cell lung cancer was validated in internal, external and prospective cohorts and outperformed single-modality models and physicians for the studied endpoint (22).

Nevertheless, prediction models remain vulnerable to selection bias, treatment-confounder effects, inconsistent endpoints and optimism from repeated feature selection. Radiomic features are sensitive to segmentation, reconstruction, voxel size and harmonization. Further work therefore calls for standardized acquisition, transparent preprocessing, calibration, external validation and clinically meaningful decision analysis (39, 40).

### 3.4 Reusable representations and foundation-model pretraining

The technical transition is enabled by three ideas. First, self-supervised learning extracts supervisory signals from the images themselves through masking, reconstruction, contrastive learning or temporal consistency (8, 14, 15). Second, language provides scalable semantic supervision because reports contain anatomy, findings, comparisons and diagnostic impressions (6, 7, 16, 17). Third, parameter-efficient adaptation allows a shared encoder to support new tasks with fewer labeled examples (5, 16, 17).

Generic computer-vision milestones shaped medical development. Contrastive language-image pretraining aligned visual and text embeddings at scale, while the Segment Anything Model introduced promptable segmentation. Medical adaptations show both the promise and limits of transfer. MedSAM was trained on more than 1.5 million image-mask pairs across 10 modalities and demonstrated broad segmentation capability; however, independent evaluations of generic SAM found reduced performance for low-contrast or poorly bounded medical targets (41–44).

Recent evidence also emphasizes the importance of modality-aligned pretraining. Dual-DINOv3 independently pretrained PET- and CT-specific self-supervised encoders and fused their representations through multiscale cross-attention for PET/CT tumor segmentation. Evaluation on the HECKTOR benchmark showed that natural-image initialization degraded performance relative to training from scratch, whereas domain-aligned CT pretraining improved segmentation and dual-stream PET/CT pretraining achieved the best overall performance. Although the authors describe the framework as a PET/CT foundation model, only one downstream task was evaluated; under the operational taxonomy proposed in Table 2, it is therefore classified conservatively as a task-specific segmentation model using reusable modality-aligned representations (45).

**Table 2:** Operational taxonomy of PET/CT foundation-model systems. Classes are assigned according to demonstrated capabilities rather than model size.

Class	Operational definition	Minimum evidence for classification
T0: Task-specific or transfer model	Optimized for one endpoint or a tightly defined task family. It may use self-supervised pretraining or transfer learning, but broad downstream reuse has not been demonstrated.	Evaluation on the intended task; no cross-task transfer is required. A large or pretrained backbone alone is insufficient for foundation-model designation.
F1: Reusable representation foundation model	A shared PET, CT, or PET/CT encoder is pretrained on broad data and reused through probing, adapters, parameter-efficient tuning, or fine-tuning.	Transfer to at least two downstream tasks or materially distinct domains, with evidence of label efficiency, transfer benefit, or improvement over training from scratch.
F2: Promptable foundation model	A shared pretrained model changes its target or output through point, box, mask, text, or other prompts without full task-specific retraining.	Evaluation across at least two target types, including an unseen or out-of-domain target when feasible; prompt sensitivity, interaction burden, and failure modes should be reported.
F3: Multimodal or vision-language foundation model	PET or PET/CT is aligned with reports, clinical text, structured records, or other modalities within a reusable cross-modal representation.	At least two cross-modal capabilities, such as grounding, retrieval, classification, question answering, or generation; evaluation should address traceability, quantitative correctness, and missing-modality behavior.
F4: Generalist clinical foundation model	One shared model core supports at least three clinically distinct task families, such as segmentation, detection, quantification, retrieval, prediction, and reporting.	Cross-task and external evaluation; assessment of cross-output consistency, degraded or missing modalities, uncertainty, and abstention behavior.
A: Agentic foundation-model system	A language or multimodal model orchestrates independently testable tools for image processing, quantification, retrieval, interpretation, and reporting. This is a system-level rather than model-level category.	Tool-level validation, provenance, versioning, error handling, audit logs, and end-to-end workflow evaluation. Orchestration alone does not make the underlying system a PET/CT foundation model.

The classes are capability categories rather than ordinal quality levels. F2 and F3 are nonsequential, and the agentic category is orthogonal because an agentic system may orchestrate task-specific tools, foundation models, or both. Capability class should be reported separately from evidence maturity and clinical readiness.

Vision-language foundation models extend beyond segmentation. BiomedGPT supports diverse biomedical tasks, RadFM incorporates large-scale two- and three-dimensional medical data, CT-CLIP and CT-CHAT use paired volumetric CT and reports, and Merlin aligns CT volumes with reports and structured electronic health record data. Collectively, these studies demonstrate that paired clinical text can provide scalable supervision that reduces dependence on some manually curated labels, while supporting retrieval, classification, and generative interaction. They do not establish that a generic radiology model understands tracer physiology or quantitative PET (6–9). Clinically focused applications of imaging foundation models are also emerging outside PET/CT; for example, a CT-based deep foundation model has been evaluated for prediction of immune checkpoint inhibitor–induced pneumonitis risk in lung cancer (46), illustrating the use of a foundation-model approach for a narrowly defined oncology endpoint. Figure 2 presents a proposed PET/CT foundation-model architecture that aligns molecular imaging, anatomic CT, clinical text, and longitudinal data within a shared representation while retaining modular, traceable outputs for segmentation, retrieval, quantification, response assessment, and report generation.

**Figure 2:**
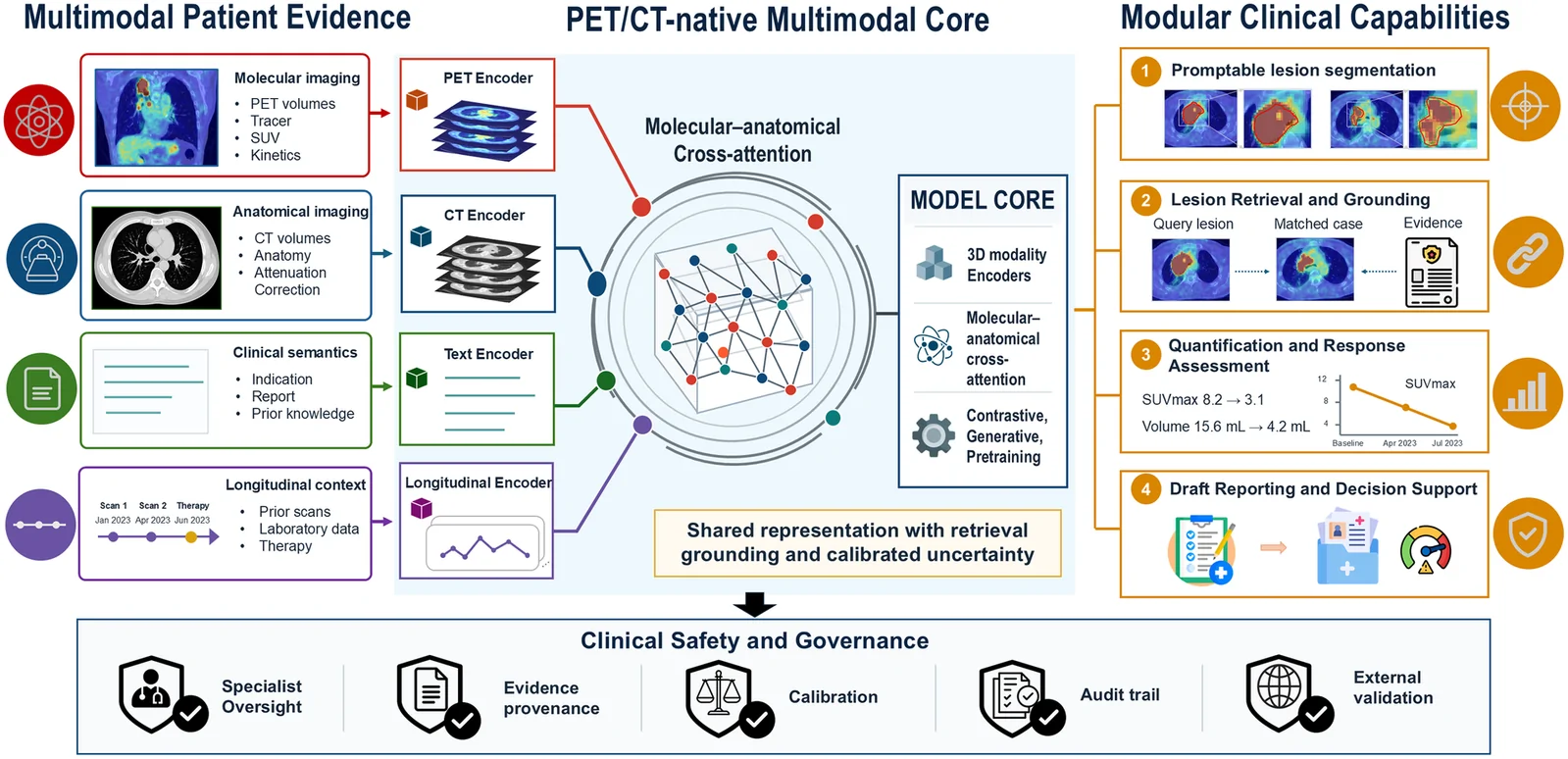
Proposed conceptual architecture for a PET/CT multimodal foundation model. PET, CT, clinical text and longitudinal data are aligned in a shared three-dimensional representation. Task outputs remain modular and are surrounded by human oversight, provenance, calibration and audit mechanisms. This architecture is conceptual and has not undergone empirical validation, including prospective clinical validation; it is intended to illustrate a potential modular design framework rather than a validated clinical system.

A useful distinction is therefore needed. A large pretrained model is not automatically a clinically useful foundation model, and a model described as generalist may remain limited to one modality or anatomic region. Foundation status should be supported by evidence of task breadth, adaptation efficiency, cross-domain transfer, robust external performance and transparent limitations. Recent medical foundation-model studies similarly warn against equating parameter count with generalizability (14–17).

### 3.5 Operational taxonomy of PET/CT foundation-model systems

For consistency, we classify PET/CT AI systems using the operational taxonomy summarized in Table 2, based on demonstrated capabilities rather than model size or terminology. The taxonomy distinguishes task-specific or transfer models, reusable pretrained representations, promptable foundation models, multimodal or vision-language foundation models, generalist clinical foundation models, and agentic systems. Capability class is considered separately from evidence maturity and clinical readiness.

## 4 PET-native multimodal and vision-language models

### 4.1 Promptable and pretrained PET representations

PET-specific foundation models began to emerge in 2025. SegAnyPET introduced a three-dimensional promptable segmentation model trained on PETS-5k (5,731 whole-body FDG PET volumes). It achieved mean Dice similarity coefficients of 90.49%–91.05% for prompted organ segmentation in its evaluated setting, including organs seen during training; performance was lower for unseen organs and markedly lower for zero-shot multifocal tumor segmentation. It should therefore be interpreted as a promptable PET representation rather than evidence of universal lesion segmentation (47). A parallel cross-modal effort, FratMAE, paired separate PET and CT vision-transformer encoders with cross-attention masked-autoencoder pretraining, providing an early demonstration that anatomic and molecular streams can be fused within a shared pretraining objective rather than concatenated *post hoc*; as a preprint focused on a defined task family, it still requires broader external and cross-tracer evaluation (48). In 2026, an open multicenter FDG PET/CT foundation model used 4,797 scans from four public datasets and held out 200 AutoPET scans for testing. With a SwinUNETR-v2-base backbone, masked-autoencoder initialization improved Dice from 0.4889 to 0.6132 with 70 labeled training scans and from 0.6414 to 0.7205 with 700; independent non-FDG and prospective validation remain absent (49). Prompt-agent reconstruction and cross-scanner diffusion denoising reuse pretrained priors but are classified conservatively as task-specific or transfer systems within this taxonomy because broad cross-task adaptation has not been demonstrated (19, 30).

### 4.2 PET/CT vision-language grounding and report generation

One of the most rapidly emerging frontiers is PET/CT vision-language modeling. PET reports contain lesion locations, tracer avidity, SUV values, comparison with prior examinations and an impression that integrates disease distribution. A model must therefore connect words to three-dimensional, often multifocal findings. ConTEXTual Net 3D used weak labels derived from report mentions and scan coordinates to train text-guided PET/CT visual grounding. This approach converts routine reports into scalable spatial supervision, although report omissions and automatically generated labels can introduce systematic bias (50).

PET2Rep established a benchmark of 565 whole-body PET/CT–report pairs and evaluated 30 general-purpose and medical vision-language models. Its best reported overall reference score was 26.8%, compared with 27.5% for a fixed-template baseline; this composite score summarizes heterogeneous language and clinical-efficacy metrics and should not be interpreted as an accuracy percentage. Practically, the result indicates that the best evaluated model did not outperform the fixed-template baseline on the benchmark's aggregate measure, underscoring limitations in the performance of currently evaluated PET/CT report-generation models (51). PETAR aligned PET, CT, lesion masks, and 11,356 localized descriptions from 5,126 examinations. PETAR-4B reported GREEN 0.257 and RaTEScore 0.713, and five physicians preferred or tied its outputs in 69 of 116 blinded comparisons. However, numeric values were masked during training and quantitative errors were excluded from the reader evaluation, limiting conclusions about SUV or lesion-size fidelity (52). PETRG-3D similarly uses separate PET and CT branches and style-adaptive prompts for a multicenter lymphoma corpus comprising 824 PET/CT reports (53).

Moving from task benchmarks toward a self-described generalist system, the SDF-HOLO used 10,460 PET/CT–report studies from four tertiary hospitals in China and combined dual-stream anatomical–metabolic encoding with voxel–text alignment for segmentation, detection, and multilingual reporting. It reported AutoPET Dice values of 0.6328–0.6933 and ROUGE-L of 0.4608 (54). A parallel 2026 pilot study used a large language model to orchestrate validated tools for end-to-end PET/CT interpretation and quantification. This agentic pattern may offer a more modular and inspectable architecture than asking a single monolithic model to perform every operation internally: segmentation, SUV measurement, structured reasoning and drafting can remain versioned, testable and replaceable. The pilot remains early evidence and should not be interpreted as support for autonomous clinical use (55). Representative studies are summarized in Table 3, which distinguishes reported quantitative performance from evidence maturity. Although several systems demonstrate strong technical performance, none has yet undergone prospective clinical-impact evaluation.

**Table 3:** Representative studies marking the transition toward PET-native foundation and vision-language models.

Study/year	Data and modality	Capability	Key contribution	Key result	Evidence gap
Leung et al., 2024 (37)	1,019 FDG/PSMA PET/CT scans	Multi-cancer segmentation and prognostic modeling	Cross-disease, cross-tracer transfer within a task family	Lesion TPR 0.77; response-prediction AUC 0.72–0.76	Retrospective; predefined endpoints
SegAnyPET, 2025 (47)	PETS-5k; 5,731 3D PET volumes	Promptable multi-organ segmentation	PET-native 3D segmentation foundation model	Mean Dice 0.90–0.91; one-prompt Dice 0.90	Independent clinical and multitracer validation
Huemann et al., 2026 (50)	25,578 exams; 11,356 weakly labeled pairs	Text-guided visual grounding	Scalable report-to-lesion supervision	F1 0.80 vs. 0.22–0.53 for baseline models	Weak-label and report-selection bias
PET2Rep, 2026 (51)	565 whole-body PET/CT image-report pairs	Benchmarking report generation	Clinically oriented PET language evaluation	Best overall reference score 26.8% (composite), below fixed-template baseline of 27.5%	Small benchmark; external languages/tracers
PETAR, 2026 (52)	PETARSeg-11K; 11,356 lesion-description pairs from >5,000 PET/CT exams	Localized lesion captioning and report generation	Mask-aware PET/CT-language alignment	GREEN 0.257; RaTEScore 0.713; ≥equivalent in 60% of comparisons	Single-system cohort; prospective workflow impact
Open multicenter PET/CT FM, 2026 (49)	4,997 FDG PET/CT scans from four public datasets	Masked pretraining and low-label segmentation	Open, multicenter PET/CT representation	Dice 0.61 with 70 labels and 0.72 with 700 labels	Mainly FDG segmentation
LLM-orchestrated agent, 2026 (55)	Real-world pilot PET/CT workflow	Tool orchestration, quantification and reporting	Modular end-to-end workflow	Primary-tumor sensitivity 100%; nodal sensitivity/specificity 84.8%/39.4%	Small pilot; safety and workflow outcomes
FratMAE, 2025 (48)	Multicenter PET/CT (lymphoma, prostate)	Cross-modal masked pretraining	Twin PET/CT masked autoencoder with cross-attention fusion	Low-label Dice 0.64 vs. 0.60; staging accuracy 0.65 vs. 0.60	Task-family evaluation
PETRG-3D, 2026 (53)	824 multicenter reports; 135 external cases	3D PET/CT report generation	Dual-stream encoding with site-adaptive prompts	ROUGE-L + 31.5%; PET-All +8.2%	Lymphoma/FDG-specific and no prospective workflow evaluation
SDF-HOLO, 2026 (54)	>10,000 total-body PET/CT with reports	Segmentation, detection, multilingual reporting	Dual-stream anatomical–metabolic generalist with blinded reader gains	Segmentation Dice 0.63–0.69; ROUGE-L 0.46	Prospective validation pending

Dataset sizes, publication status and evidence gaps reflect the cited reports as of July 11, 2026. Because these studies evaluate different tasks, datasets, endpoints, and performance metrics (e.g., Dice, F1, ROUGE-L, and GREEN), the reported values are study-specific and should not be interpreted as directly comparable measures of model performance.

### 4.3 Current evidence gaps

Four gaps are conspicuous. First, most PET foundation models focus on FDG oncology; evidence for neurodegenerative, cardiac, pediatric, infection/inflammation and theranostic applications is sparse. Second, longitudinal reasoning is underdeveloped even though response assessment depends on consistent comparison across time. A longitudinally aware network for pediatric Hodgkin lymphoma shows the task-specific potential of serial modeling, but generalist longitudinal PET benchmarks remain absent (56). Newer resources are beginning to close the report-linked gap; for example, PETWB-REP releases whole-body FDG PET/CT from 490 patients spanning multiple cancers with paired radiology reports, although it remains single-institution and FDG-only (57). Table 4 maps the principal datasets and benchmarks supporting this emerging ecosystem and highlights the limited tracer diversity, longitudinal linkage and report-level supervision available for foundation-model evaluation.

**Table 4:** Key datasets and benchmarks supporting the PET/CT foundation-model ecosystem, with their principal uses and current limitations.

Resource	Scale/dataset	Primary use	Limitation for foundation-model evaluation
FDG-PET/CT-Lesions/autoPET (32, 35)	1,014 whole-body FDG PET/CT studies with lesion annotations	Whole-body lesion segmentation	Single dominant tracer and defined tumor categories
HECKTOR (33, 34)	Multicenter head-and-neck FDG PET/CT with tumor/nodal labels and outcomes	Segmentation and outcome prediction	Regional disease focus; not report aligned
PETS-5k initial release (47)	5,731 whole-body 3D PET volumes	Promptable multi-organ segmentation (five annotated organs)	Annotation quality heterogeneity; limited public clinical metadata
PET2Rep (51)	565 whole-body PET/CT report pairs	Whole-body report-generation benchmark	Limited scale for broad generalization claims
PETARSeg-11K (52)	>11,000 lesion descriptions and masks from >5,000 exams	Localized finding generation	Derived labels and single-system practice patterns
CT-RATE (8)	25,692 chest CT scans paired with reports	3D contrastive learning, retrieval and chat	No molecular imaging signal; transfer to PET unproven
Merlin dataset (9)	15,331 abdominal CT scans plus reports and EHR codes	3D multimodal pretraining	CT-centric; tracer physiology absent
PETWB-REP (57)	490 multi-cancer whole-body FDG PET/CT with reports	Report-linked multimodal learning	Single-institution; FDG only

Third, the relationship between text generation and quantitative correctness is unresolved (50–53). A report may correctly mention a lesion but misstate its SUV, size, temporal change or anatomic location. Fourth, prospective human-AI evidence is rare. The field has many retrospective accuracy studies but few silent deployments, crossover reader trials, time-motion studies or patient-outcome evaluations (13, 55, 56).

## 5 Clinical opportunities and translation challenges

The opportunities outlined below remain prospective and should not be interpreted as established clinical benefits; their translation into routine practice will require task-appropriate external validation and, where relevant, prospective clinical evaluation.

### 5.1 Acquisition and quantitative image formation

A pretrained molecular-imaging encoder could adapt to low-count denoising, reconstruction, motion correction and quality control across scanners and protocols. Total-body PET may provide a useful setting for representation learning because it produces high-sensitivity dynamic and whole-body data at a scale that could support such approaches. Potential clinical benefits could include lower activity, faster scans, and improved access. A key translational requirement is preservation of quantitative fidelity at lesion, organ and kinetic levels (2, 27, 28).

### 5.2 Universal segmentation and tumor-burden phenotyping

Promptable segmentation may enable automatic models to function as interactive physician tools (42–44). A user could specify a lesion, organ or physiologic region and obtain an editable three-dimensional contour, rather than accepting a fixed tumor mask. Shared representations could also support lesion tracking, total metabolic tumor volume, organ-specific disease burden, and radiopharmaceutical dosimetry. The interface should expose uncertainty and any gains in correction efficiency should be evaluated against manual contouring.

### 5.3 Reporting, retrieval and longitudinal comparison

Vision-language alignment may support retrieval of similar cases, grounding of report phrases to lesions, structured draft generation, and automated comparison with prior scans (5, 6, 50, 52). For response assessment, the system should pair each current lesion with its prior counterpart, calculate quantitative change, identify new lesions and generate an evidence-linked draft. Every generated statement should be traceable to an image region, quantitative tool output or clinical source.

### 5.4 Theranostics and precision oncology

Nuclear medicine uniquely connects diagnosis and therapy. A multimodal model could integrate target-expression PET, tumor burden, organ dosimetry, laboratory trends, prior treatments and outcomes to support patient selection and treatment planning. Such systems could help standardize complex workflows, but causal treatment recommendations require stronger evidence than diagnostic assistance. Retrospective associations should not be presented as evidence that a model can select the best radiopharmaceutical or activity.

### 5.5 Agentic orchestration as a near-term architecture

The near-term clinical system is likely to be a supervised orchestrator rather than an autonomous foundation model. A language model can route a case through DICOM validation, registration, organ and lesion segmentation, SUV extraction, response rules, retrieval and report drafting. Each tool can retain its own version, validation record and failure checks. The nuclear medicine physician remains responsible for reconciling discordant evidence and approving the report. Early PET/CT agent evidence supports feasibility but not autonomous deployment (55).

### 5.6 Risks and unresolved technical problems

#### 5.6.1 Hallucination and semantic overconfidence

Medical vision-language models can generate convincing but unsupported details. In one evaluation of GPT-4V across radiologic images, false-positive rates were high and performance for the tasks studied remained well below expert readers. PET reporting adds further opportunities for hallucination through tracer name, SUV, lesion count, comparison and staging language. A clinically safe model should abstain, cite its evidence and separate measured facts from inferred impressions (13).

#### 5.6.2 Quantitative drift hidden by visual quality

A foundation model may adapt well to classification while altering SUV distributions or lesion texture (11, 12, 36). Quantitative drift can result from scanner upgrades, reconstruction changes, dose protocols or model updates (10). PET validation should include Bland-Altman analysis, repeatability coefficients, lesion-size strata, high- and low-uptake lesions, physiologic reference organs and response-category concordance.

#### 5.6.3 Cross-tracer negative transfer

Pooling tracers without explicit conditioning can create negative transfer (26, 27). A model may learn that intense salivary-gland uptake is suspicious from one training context and normal from another or conflate treatment-related and pathologic patterns. Tracer identity, injected activity, uptake interval and acquisition protocol should be explicit inputs (49). Evaluation should report performance by tracer and by tracer-disease combination, including rare combinations.

#### 5.6.4 Dataset leakage and report-derived label bias

Foundation-model corpora create new leakage pathways (14–17). The same patient may appear across serial scans, public dataset derivatives or training and evaluation reports. Report-derived labels also reproduce the omissions, hedging, stylistic variation and prior assumptions of the reporting practice. Patient-level and institution-level deduplication, temporal splits, source provenance and contamination audits are essential.

#### 5.6.5 Fairness, privacy and access

Scanner availability, tracer access and referral patterns vary across health systems. A model trained at tertiary cancer centers may underperform in community practice, in pediatric patients or in populations with different disease prevalence. Large multimodal data also increases re-identification risk because images, text and longitudinal metadata can be linked. Federated learning, secure computation and de-identification can reduce some risks, but governance must define permissible use, access, retention and accountability (38, 58).

## 6 MOSAIC-PET framework and translational roadmap

### 6.1 MOSAIC-PET clinical-readiness framework

We propose MOSAIC-PET as an author-developed, task-agnostic framework for evaluating PET/CT foundation models across reconstruction, segmentation, retrieval, reporting, prediction, and agentic workflows. It is intended to complement, rather than replace, CLAIM 2024 for imaging-AI reporting, TRIPOD + AI for prediction models, FUTURE-AI for trustworthy lifecycle principles, DECIDE-AI for early live clinical evaluation, REAL-FM for evaluating biomedical foundation models claims, and REFINE for foundation- and large-language-model reporting (58–63). MOSAIC-PET adds PET-specific requirements for quantitative molecular fidelity, image–text grounding, tracer-conditioned adaptation, clinical integration, and lifecycle control. Its six domains are a structured synthesis of the reviewed literature and the authors' judgment; they have not undergone Delphi consensus, weighting, threshold validation, or prospective testing. The framework must therefore not be used as a certification score. Figure 3 introduces the domains, and Table 5 translates them into intended-use-specific questions, minimum evaluations, and illustrative stop criteria.

**Figure 3:**
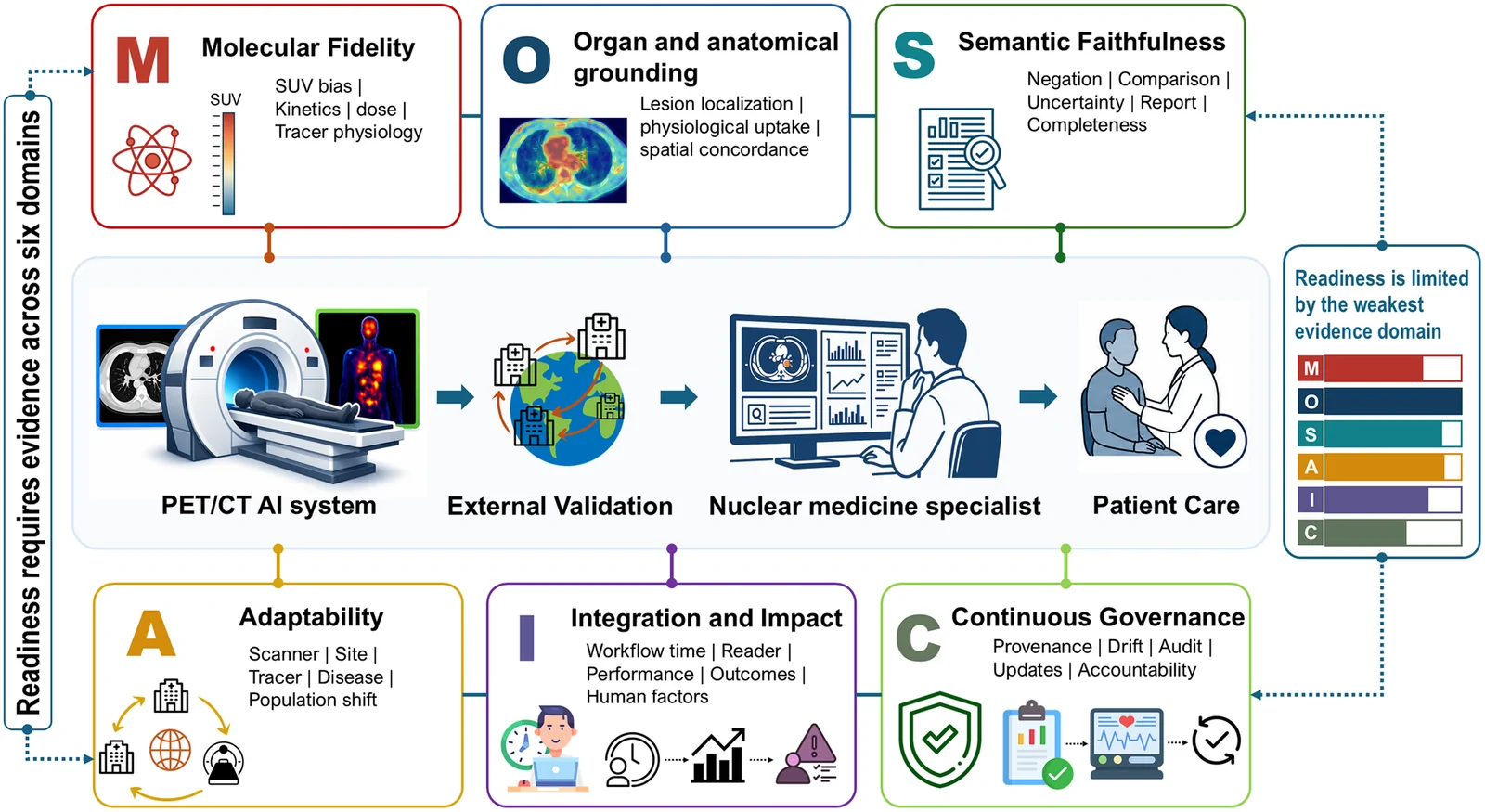
The proposed MOSAIC-PET evaluation framework. Each letter defines a PET-specific evidence domain. Advancement should depend on balanced evidence across all domains rather than a single aggregate accuracy or language metric.

**Table 5:** The proposed operationalization of MOSAIC-PET.

Domain	Core question	Minimum evaluation	Example stop criterion
M: Molecular fidelity	Does the model preserve the physical and biologic meaning of PET?	SUV bias and limits of agreement; lesion/organ kinetics; dose and count strata; response concordance	Clinically material SUV bias or loss of small/low-uptake lesions
O: Organ/anatomic grounding	Are findings correctly localized and separated from physiologic uptake?	Lesion-level sensitivity/false positives; spatial overlap; organ-specific errors; misregistration stress tests	High false-positive burden in physiologic organs or wrong-site grounding
S: Semantic faithfulness	Is every generated statement supported?	Expert factuality; negation, uncertainty, comparison and number accuracy; image-text traceability	Unsupported staging, lesion, SUV or temporal claim
A: Adaptability	Does performance survive real domain shifts?	External sites; vendors; protocols; tracers; diseases; demographic and prevalence subgroups	Material degradation in a prespecified subgroup or tracer
I: Integration and impact	Does the system improve care or workflow?	Silent deployment; reader study; calibration; time, correction burden, computational/resource requirements, and decision impact	No net time benefit, automation bias or worse reader performance
C: Continuous governance	Can changes and failures be detected and controlled?	Versioning; provenance; drift monitoring; audit logs; rollback; update plan	Undetectable drift, irreproducible output or unclear accountability

The domains, minimum evaluations and example stop criteria are intended as a proposed evaluation framework; thresholds should be prespecified according to the intended use and clinical risk.

### 6.2 Translational roadmap

Figure 4 summarizes five macro-stages from broad PET/CT pretraining to clinical impact: build the data foundation, define the intended use, test clinically meaningful performance, deploy progressively, and govern the total product lifecycle. Sections 6.2.1–6.2.6 provide six implementation actions mapped to those five stages; human-factors and equitable-implementation validation is separated as a cross-cutting action spanning clinical testing and progressive deployment. Accumulating evidence and continuous monitoring connect the stages, emphasizing that scale alone does not establish clinical readiness.

**Figure 4:**
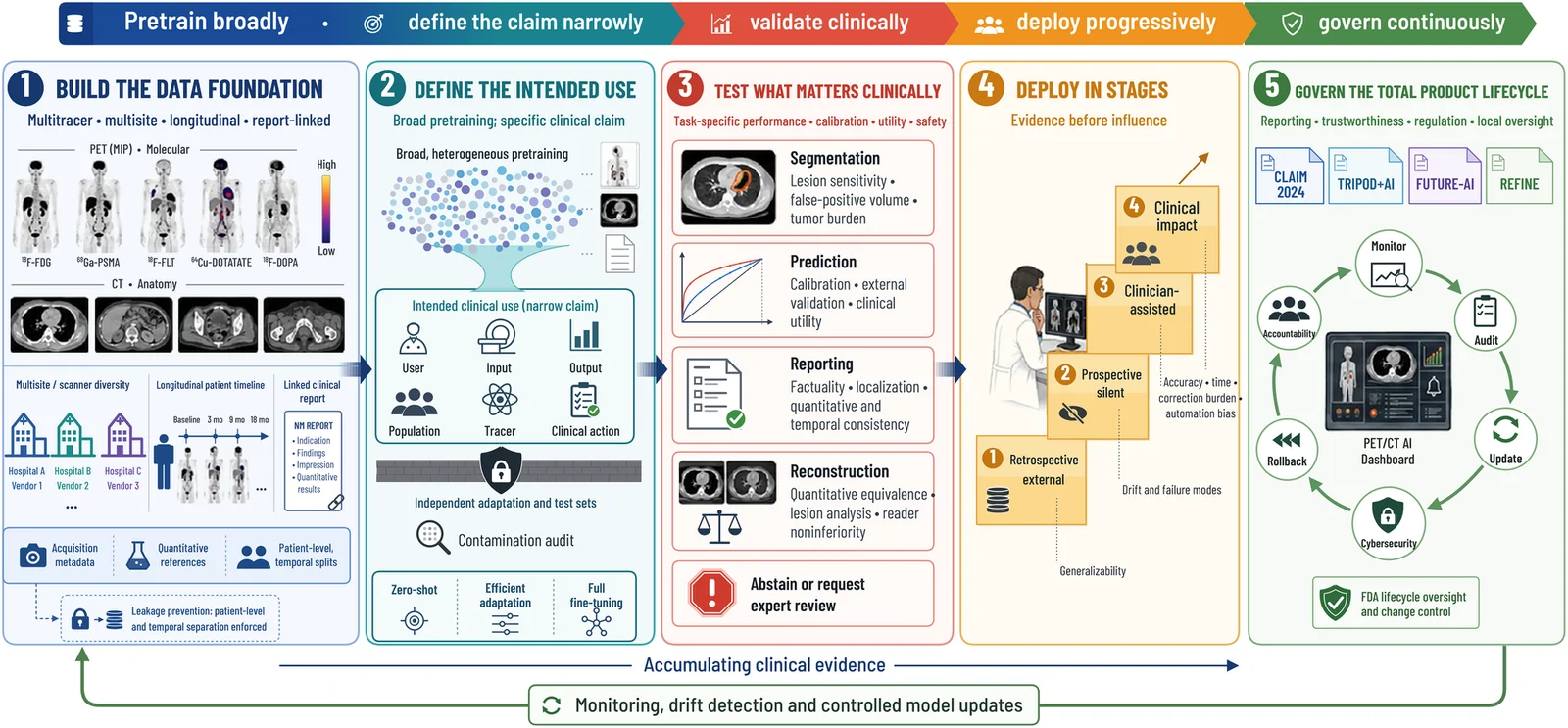
From foundation model to clinical impact: a translational roadmap for PET/CT AI. The roadmap links five stages: building multi-tracer, multisite, longitudinal and report-linked data; defining a narrow intended use; generating clinically weighted evidence; progressing through retrospective, silent, assisted and clinical-impact evaluation; and maintaining continuous lifecycle governance. Broad pretraining is only the starting point; translation requires claim-specific evidence, specialist-supervised deployment and controlled model updates.

#### 6.2.1 Build multi-tracer, longitudinal and report-linked corpora

The field requires datasets that span institutions, scanner vendors, reconstruction methods, disease states, and radiotracers. Each dataset should include de-identified acquisition metadata, injected activity, uptake time, reconstruction and correction parameters, quantitative reference regions, lesion annotations where available, clinical reports, and relevant outcomes. Longitudinal examinations should be linked at the patient level, with data partitioned to prevent patient-level and temporal leakage. Public challenges have demonstrated how shared benchmarks can accelerate segmentation research; the next generation should incorporate report-linked data, serial examinations, and multi-tracer diversity (32, 33, 35).

#### 6.2.2 Separate pretraining breadth from intended-use evaluation

A foundation model may be pretrained on heterogeneous data, but its regulatory and clinical claims should remain specific to the intended use (58, 61, 63). Developers should clearly define the intended user, inputs, outputs, target population, radiotracer, workflow position, and supported clinical action. Adaptation and testing datasets must remain independent of pretraining data, and potential data contamination should be systematically assessed and reported. Evidence should also distinguish among zero-shot performance, parameter-efficient adaptation, and full model fine-tuning.

#### 6.2.3 Evaluate clinically meaningful performance, safety, and utility

Dice coefficient, AUROC, BLEU and ROUGE provide useful technical information but are insufficient to establish clinical value. Lesion-segmentation studies should assess false-positive volume, lesion-level sensitivity and agreement in total tumor burden (35, 36, 47, 64). Prediction models require calibration, external testing and clinical-utility analysis (22, 37, 59). Report-generation systems should be evaluated for factual accuracy, completeness, anatomic localization, quantitative correctness, negation, uncertainty and longitudinal consistency (50–52, 55). Reconstruction, enhancement, and generative image-synthesis models require quantitative equivalence, lesion-level analysis, and reader-assessed noninferiority (10–12, 21). Evaluation datasets should also include challenging cases in which the model is expected to abstain or request expert review (58, 63).

#### 6.2.4 Validate human factors, workflow integration, and equitable implementation

Clinical translation requires evidence that extends beyond standalone model performance. Human-factors studies should evaluate usability, interpretability, alert and correction burden, automation bias, appropriate reliance, and the ability of clinicians to recognize and override erroneous outputs. Evaluation should include nuclear medicine physicians with different experience levels and should examine whether performance varies across institutions, demographic groups, disease presentations, radiotracers, scanner platforms, and resource settings. Implementation studies should additionally assess interoperability with PACS, reporting systems, and clinical workflows; cybersecurity requirements; staff training; and cost-effectiveness. These considerations are particularly important for foundation models because their broad capabilities may create new forms of interaction, overreliance, and performance disparity that are not captured by conventional retrospective benchmarks (58–61).

Computational feasibility should also be considered in assessments of clinical readiness. Training, adapting, or locally deploying large multimodal foundation models may require substantial accelerator hardware and memory, storage capacity, network bandwidth, and technical support, which may limit adoption in smaller or resource-constrained nuclear medicine services. Centralized or cloud-based inference may reduce local hardware requirements but may introduce dependencies on connectivity, latency, data governance, and recurring infrastructure costs (65). Accordingly, translational evaluations should report hardware requirements, inference time, storage and memory demands, deployment architecture, and maintenance burden, and should consider parameter-efficient adaptation, model compression, or modular workflows when these can achieve the intended clinical function with lower computational requirements (5).

#### 6.2.5 Progress from silent evaluation to assisted use and clinical-impact studies

A rational deployment pathway should begin with retrospective external testing, followed by prospective silent evaluation to characterize real-world performance, data drift, and failure modes without influencing clinical decisions (58, 66). The subsequent assisted phase should assess reader accuracy, interpretation time, correction burden, and automation bias through crossover or randomized studies. Only after these requirements are satisfied should studies investigate effects on clinical management, patient outcomes, and resource utilization. Performance monitoring must continue after deployment because scanners, radiotracers, acquisition protocols, and patient populations change over time.

#### 6.2.6 Align reporting, trustworthiness and regulation

PET/CT foundation-model studies should use CLAIM 2024 for imaging-AI reporting, TRIPOD + AI when a prediction model is developed or evaluated, FUTURE-AI for trustworthy lifecycle principles, REFINE for foundation- and large-language-model reporting, and DECIDE-AI for early-stage prospective evaluation in live clinical settings (58–62). In the United States, the FDA's January 2025 draft guidance describes lifecycle-management and marketing-submission recommendations for AI-enabled device software functions; it remains draft guidance. The FDA's August 2025 final guidance on predetermined change control plans addresses prespecified modifications to AI-enabled device software functions (66, 67). Regulatory submissions and institutional governance should therefore address intended use, update boundaries, performance monitoring, cybersecurity, clinician- and patient-facing information, auditability, and rollback procedures rather than treating authorization as a one-time event. Figure 3, Table 5, and Figure 4 connect PET/CT-specific evidence domains with claim-specific testing, specialist-supervised deployment, and lifecycle control.

Patient-level transparency should accompany technical and regulatory governance. When AI meaningfully contributes to image interpretation, quantitative assessment, or clinical decision support, patients should receive information appropriate to the intended use regarding the role of AI, the continued responsibility of the clinical team, and relevant limitations or uncertainties. Requirements for specific consent or notification may vary according to the clinical context, jurisdiction, institutional policy, secondary use of patient data for model development or evaluation, and the degree of system autonomy. Institutions should therefore define how patient-facing information is communicated, how questions or concerns are addressed, and, where applicable, how consent or opt-out mechanisms are implemented. These considerations should be incorporated into implementation and governance planning alongside privacy, safety, performance monitoring, and accountability.

### 6.3 Limitations of this review

This review has several limitations. First, it was designed as a focused narrative review rather than a systematic review; therefore, the search strategy was not intended to achieve exhaustive study capture or formal risk-of-bias assessment. Second, the search was restricted to English-language literature identified through PubMed, arXiv, and official conference proceedings repositories, potentially excluding relevant studies indexed elsewhere or published in other languages. Third, given the rapidly evolving nature of PET/CT foundation-model research, selected recent preprints were included. These studies were explicitly identified as preprints and interpreted as preliminary evidence, as their findings may change following peer review. Fourth, general medical-imaging foundation-model studies were included selectively when they provided directly relevant methodological or translational context, which necessarily introduced an element of author judgment. Finally, the literature search covered studies available through July 11, 2026; subsequent developments are therefore not represented. Accordingly, this review should be interpreted as a structured synthesis of the contemporary evidence landscape rather than an exhaustive or formally graded systematic review.

## 7 Conclusions and future directions

The reviewed evidence supports a transition in development strategy, not yet a transition to routine clinical practice. Task-specific PET/CT systems have established technical feasibility for reconstruction, enhancement, segmentation, quantification, and outcome modeling. More recent promptable, pretrained, vision-language, and agentic systems broaden the range of reusable components, but the evidence base is heterogeneous and dominated by retrospective cohorts, task-specific endpoints, FDG oncology, and single-system datasets. Technical performance across Dice, F1, language metrics, reader ratings, and staging measures is not directly comparable and does not by itself demonstrate clinical utility. The most defensible near-term architecture is therefore modular and claim-specific: PET physics, quantitative tools, image–text grounding, and language generation should remain separately testable, evidence linked, and overseen by nuclear medicine specialists. This is a reasoned translational proposal, not a prediction validated by comparative deployment trials. Future work should prioritize multitracer and longitudinal PET/CT corpora with acquisition metadata and institutionally sequestered test sets; PET-specific factuality benchmarks for tracer identity, localization, lesion count, SUV, size, negation, uncertainty, and temporal change; and prospective studies that progress from silent evaluation to reader-assistance and clinical-impact endpoints. Dynamic and total-body PET, theranostic dosimetry, human factors, interoperability, compute burden, and post-deployment drift require explicit evaluation. A credible PET/CT foundation-model ecosystem should preserve quantitative molecular meaning, expose uncertainty, ground language in images and measurements, generalize across clinically relevant domains, and remain auditable throughout its lifecycle. MOSAIC-PET offers a proposed structure for organizing that evidence, but its own clinical value must be established empirically.

## References

[1] Sitek A Ahn S Asma E Chandler A Ihsani A Prevrhal S. Artificial intelligence in PET: an industry perspective. PET Clin. (2021) 16(4):483–92. 10.1016/j.cpet.2021.06.006 34353746

[2] Reader AJ Pan B. AI for PET image reconstruction. Br J Radiol. (2023) 96(1150):20230292. 10.1259/bjr.20230292 37486607 PMC10546435

[3] Matsubara K Ibaraki M Nemoto M Watabe H Kimura Y. A review on AI in PET imaging. Ann Nucl Med. (2022) 36(2):133–43. 10.1007/s12149-021-01710-8 35029818

[4] Artesani A Bruno A Gelardi F Chiti A. Empowering PET: harnessing deep learning for improved clinical insight. Eur Radiol Exp. (2024) 8(1):17. 10.1186/s41747-023-00413-1 38321340 PMC10847083

[5] Muneer A Waqas M Saad MB Showkatian E Bandyopadhyay R Xu H. From classical machine learning to emerging foundation models: review on multimodal data integration for cancer research. Artif Intell Rev. (2026) 59(4):119. 10.1007/s10462-026-11522-9 PMC13664680 42877340

[6] Zhang K Zhou R Adhikarla E Yan Z Liu Y Yu J. A generalist vision-language foundation model for diverse biomedical tasks. Nat Med. (2024) 30(11):3129–41. 10.1038/s41591-024-03185-2 39112796 PMC12581140

[7] Wu C Zhang X Zhang Y Hui H Wang Y Xie W. Towards generalist foundation model for radiology by leveraging web-scale 2D&3D medical data. Nat Commun. (2025) 16(1):7866. 10.1038/s41467-025-62385-7 40849424 PMC12375113

[8] Hamamci IE Er S Wang C Almas F Simsek AG Esirgun SN. Generalist foundation models from a multimodal dataset for 3D computed tomography. Nat Biomed Eng. (2026) 10:1610–28. 10.1038/s41551-025-01599-y 41680439 PMC13658014

[9] Blankemeier L Kumar A Cohen JP Liu J Liu L Van Veen D. Merlin: a computed tomography vision-language foundation model and dataset. Nature. (2026) 652(8112):1318–28. 10.1038/s41586-026-10181-8 41781626 PMC13082451

[10] Zaidi H El Naqa I. Quantitative molecular positron emission tomography imaging using advanced deep learning techniques. Annu Rev Biomed Eng. (2021) 23:249–76. 10.1146/annurev-bioeng-082420-020343 33797938

[11] Chaudhari AS Mittra E Davidzon GA Gulaka P Gandhi H Brown A. Low-count whole-body PET with deep learning in a multicenter and externally validated study. npj Digit Med. (2021) 4:127. 10.1038/s41746-021-00497-2 34426629 PMC8382711

[12] Hashimoto F Onishi Y Ote K Tashima H Reader AJ Yamaya T. Deep learning-based PET image denoising and reconstruction: a review. Radiol Phys Technol. (2024) 17(1):24–46. 10.1007/s12194-024-00780-3 38319563 PMC10902118

[13] Strotzer QD Nieberle F Kupke LS Napodano G Muertz AK Meiler S. Toward foundation models in radiology? Quantitative assessment of GPT-4V’s multimodal and multianatomic region capabilities. Radiology. (2024) 313(2):e240955. 10.1148/radiol.240955 39589253

[14] Huang SC Jensen M Yeung-Levy S Lungren MP Poon H Chaudhari AS. A systematic review and implementation guidelines of multimodal foundation models in medical imaging. Res Sq. (2025). 10.21203/rs.3.rs-5537908/v1

[15] Bian Y Li J Ye C Jia X Yang Q. Artificial intelligence in medical imaging: from task-specific models to large-scale foundation models. Chin Med J. (2025) 138(6):651–63. 10.1097/CM9.0000000000003489 40008785 PMC11925424

[16] Ryu JS Kang H Chu Y Yang S. Vision-language foundation models for medical imaging: a review of current practices and innovations. Biomed Eng Lett. (2025) 15(5):809–30. 10.1007/s13534-025-00484-6 40917147 PMC12411343

[17] Zhao Z Liu Y Wu H Wang M Li Y Wang S. CLIP in medical imaging: a survey. Med Image Anal. (2025) 102:103551. 10.1016/j.media.2025.103551 40127590

[18] Xing Y Qiao W Wang T Wang Y Li C Lv Y. Deep learning-assisted PET imaging achieves fast scan/low-dose examination. EJNMMI Phys. (2022) 9(1):7. 10.1186/s40658-022-00431-9 35122172 PMC8816983

[19] Yu B Ozdemir S Dong Y Shao W Pan T Shi K. Robust whole-body PET image denoising using 3D diffusion models: evaluation across various scanners, tracers, and dose levels. Eur J Nucl Med Mol Imaging. (2025) 52(7):2549–62. 10.1007/s00259-025-07122-4 39912940 PMC12119227

[20] Peng Z Zhang F Jiang H Liu G Sun J Du Y. Deep learning-based low count whole-body positron emission tomography denoising incorporating computed tomography priors. Quant Imaging Med Surg. (2024) 14(12):8140–54. 10.21037/qims-24-489 39698633 PMC11652058

[21] Akita R Takauchi K Ishibashi M Kondo S Ono S Yokomachi K. 18F-FDG dose reduction using deep learning-based PET reconstruction. EJNMMI Res. (2025) 15(1):78. 10.1186/s13550-025-01269-9 40591189 PMC12214151

[22] Zhong Y Cai C Chen T Gui H Deng J Yang M. PET/CT based cross-modal deep learning signature to predict occult nodal metastasis in lung cancer. Nat Commun. (2023) 14(1):7513. 10.1038/s41467-023-42811-4 37980411 PMC10657428

[23] Zhao F Li D Luo R Liu M Jiang X Hu J. Self-supervised deep learning for joint 3D low-dose PET/CT image denoising. Comput Biol Med. (2023) 165:107391. 10.1016/j.compbiomed.2023.107391 37717529

[24] Spadea MF Maspero M Zaffino P Seco J. Deep learning based synthetic-CT generation in radiotherapy and PET: a review. Med Phys. (2021) 48(11):6537–66. 10.1002/mp.15150 34407209

[25] Shiyam Sundar LK Muzik O Buvat I Bidaut L Beyer T. Potentials and caveats of AI in hybrid imaging. Methods. (2021) 188:4–19. 10.1016/j.ymeth.2020.10.004 33068741

[26] Lindgren Belal S Frantz S Minarik D Enqvist O Wikström E Edenbrandt L. Applications of artificial intelligence in PSMA PET/CT for prostate cancer imaging. Semin Nucl Med. (2024) 54(1):141–9. 10.1053/j.semnuclmed.2023.06.001 37357026

[27] Zhang Q Huang Z Jin Y Li W Zheng H Liang D. Total-body PET/CT: a role of artificial intelligence? Semin Nucl Med. (2025) 55(1):124–36. 10.1053/j.semnuclmed.2024.09.002 39368911

[28] Sun Y Cheng Z Qiu J Lu W. Performance and application of the total-body PET/CT scanner: a literature review. EJNMMI Res. (2024) 14(1):38. 10.1186/s13550-023-01059-1 38607510 PMC11014840

[29] Fallahpoor M Chakraborty S Pradhan B Faust O Barua PD Chegeni H. Deep learning techniques in PET/CT imaging: a comprehensive review from sinogram to image space. Comput Methods Programs Biomed. (2024) 243:107880. 10.1016/j.cmpb.2023.107880 37924769

[30] Xie X Zhao W Nan M Zhang Z Wu Y Zheng H. Prompt-agent-driven integration of foundation model priors for low-count PET reconstruction. IEEE Trans Med Imaging. (2025) 44(10):4073–86. 10.1109/TMI.2025.3527155 40030949

[31] Huang B Chen K Li B Liu H Liu Q. ALL-PET: A low-resource and low-shot PET foundation model in projection domain. arXiv preprint arXiv:2509.09130. (2025).

[32] Gatidis S Hepp T Früh M La Fougère C Nikolaou K Pfannenberg C. A whole-body FDG-PET/CT dataset with manually annotated tumor lesions. Sci Data. (2022) 9(1):601. 10.1038/s41597-022-01718-3 36195599 PMC9532417

[33] Oreiller V Andrearczyk V Jreige M Boughdad S Elhalawani H Castelli J. Head and neck tumor segmentation in PET/CT: the HECKTOR challenge. Med Image Anal. (2022) 77:102336. 10.1016/j.media.2021.102336 35016077

[34] Andrearczyk V Oreiller V Abobakr M Akhavanallaf A Balermpas P Boughdad S. Overview of the HECKTOR Challenge at MICCAI 2022: Automatic Head and Neck Tumor Segmentation and Outcome Prediction in PET/CT. Head and Neck Tumor Segmentation and Outcome Prediction: Third Challenge, HECKTOR 2022. Lecture Notes in Computer Science. (2023). vol. 13626, p. 1–30. 10.1007/978-3-031-27420-6_1 PMC10171217 37195050

[35] Gatidis S Früh M Fabritius MP Gu S Nikolaou K Fougère CL. Results from the autoPET challenge on fully automated lesion segmentation in oncologic PET/CT imaging. Nat Mach Intell. (2024) 6:1396–405. 10.1038/s42256-024-00912-9

[36] Shiyam Sundar LK Beyer T. Is automatic tumor segmentation on whole-body 18F-FDG PET images a clinical reality? J Nucl Med. (2024) 65(7):995–7. 10.2967/jnumed.123.267183 38844359 PMC11218718

[37] Leung KH Rowe SP Sadaghiani MS Leal JP Mena E Choyke PL. Deep semisupervised transfer learning for fully automated whole-body tumor quantification and prognosis of cancer on PET/CT. J Nucl Med. (2024) 65(4):643–50. 10.2967/jnumed.123.267048 38423786 PMC10995523

[38] Shiri I Razeghi B Vafaei Sadr A Amini M Salimi Y Ferdowsi S. Multi-institutional PET/CT image segmentation using federated deep transformer learning. Comput Methods Programs Biomed. (2023) 240:107706. 10.1016/j.cmpb.2023.107706 37506602

[39] Hatt M Cheze Le Rest C Antonorsi N Tixier F Tankyevych O Jaouen V. Radiomics in PET/CT: current status and future AI-based evolutions. Semin Nucl Med. (2021) 51(2):126–33. 10.1053/j.semnuclmed.2020.09.002 33509369

[40] Wei L El Naqa I. Artificial intelligence for response evaluation with PET/CT. Semin Nucl Med. (2021) 51(2):157–69. 10.1053/j.semnuclmed.2020.10.003 33509372 PMC8099153

[41] Radford A Kim JW Hallacy C. Learning transferable visual models from natural language supervision. In: Proceedings of the 38th International Conference on Machine Learning (PMLR) (2021).

[42] Kirillov A Mintun E Ravi N. Segment anything. In: Proceedings of the IEEE/CVF International Conference on Computer Vision (2023).

[43] Ma J He Y Li F Han L You C Wang B. Segment anything in medical images. Nat Commun. (2024) 15(1):654. 10.1038/s41467-024-44824-z 38253604 PMC10803759

[44] Mazurowski MA Dong H Gu H Yang J Konz N Zhang Y. Segment anything model for medical image analysis: an experimental study. Med Image Anal. (2023) 89:102918. 10.1016/j.media.2023.102918 37595404 PMC10528428

[45] Lin Y-N Wang S Safari M Yang X. Beyond Natural Images: A Dual-Stream DINOv3 Framework for PET/CT Segmentation. Medical Imaging with Deep Learning (2026).

[46] Muneer A Showkatian E Kitsel Y Saad MB Sujit SJ Soto F. CT-based deep foundation model for predicting immune checkpoint inhibitor-induced pneumonitis risk in lung cancer. J Immunother Cancer. (2026) 14(9):e015014. 10.1136/jitc-2026-015014 42759982 PMC13599922

[47] Zhang Y Xue L Zhang W Li L Liu Y Jiang C. SegAnyPET: universal promptable segmentation from positron emission tomography images. In: Proceedings of the IEEE/CVF International Conference on Computer Vision (2025).

[48] Oh Y Seifert R Cao Y Clement C Ferdinandus J Lapa C. Developing a PET/CT foundation model for cross-modal anatomical and functional imaging. arXiv. (2025).

[49] Liu X Zhang Q Marin T Xia M Liu C El Fakhri G. An open multicenter whole-body FDG PET/CT foundation model for tumor segmentation. arXiv. (2026).

[50] Huemann Z Church S Warner JD Tran D Tie X McMillan AB. ConTEXTual net 3D: vision-language modeling in PET/CT for visual grounding of positive findings. J Imaging Inf Med. (2026). 10.1007/s10278-026-01879-2 41979754

[51] Zhang Y Zhang W Ling Z. PET2Rep: towards vision-language model-driven automated radiology report generation for positron emission tomography. In: Proceedings of the AAAI Conference on Artificial Intelligence (2026).

[52] Maqbool D Lee C Huemann Z Church SD Larson ME Perlman SB. PETAR: localized findings generation with mask-aware vision-language modeling for PET automated reporting. In: Proceedings of the IEEE/CVF Conference on Computer Vision and Pattern Recognition (2026).

[53] Jiao W Shang K Li H. Vision-language models for automated 3D PET/CT report generation. In: IEEE/CVF Conference on Computer Vision and Pattern Recognition (CVPR) (2026).

[54] Chen W Wu L Lu S Sun Y Bi W Yuan Z. A generalist foundation model for total-body PET/CT enables diagnostic reporting and system-wide metabolic profiling. arXiv. (2026).

[55] Choi H Bae S Na KJ. End-to-end PET/CT interpretation and quantification with an LLM-orchestrated AI agent: a real-world pilot study. J Nucl Med. (2026). 10.2967/jnumed.126.272362 PMC13635056 42315314

[56] Tie X Shin M Lee C Perlman SB Huemann Z Weisman AJ. Automatic quantification of serial PET/CT images for pediatric hodgkin lymphoma using a longitudinally aware segmentation network. Radiol Artif Intell. (2025) 7(3):e240229. 10.1148/ryai.240229 39969278 PMC12127956

[57] Xue L Feng G Zhang W Zhang Y Li L Wang S. PETWB-REP: a multi-cancer whole-body FDG PET/CT dataset with corresponding radiology reports. Sci Data. (2026) 13:675. 10.1038/s41597-026-07058-w 41826325 PMC13125245

[58] Lekadir K Frangi AF Porras AR Glocker B Cintas C Langlotz CP. FUTURE-AI: international consensus guideline for trustworthy and deployable artificial intelligence in healthcare. Br Med J. (2025) 388:e081554. 10.1136/bmj-2024-081554 PMC11795397 39909534

[59] Collins GS Moons KGM Dhiman P Riley RD Beam AL Van Calster B. TRIPOD + AI statement: updated guidance for reporting clinical prediction models that use regression or machine learning methods. Br Med J. (2024) 385:e078378. 10.1136/bmj-2023-078378 PMC11019967 38626948

[60] Mese I D’Antonoli TA Bluethgen C Bressem K Cuocolo R Chaudhari A. Reporting checklist for foundation and large language models in medical research (REFINE): an international consensus guideline. Diagn Intervent Radiol. (2026). 10.4274/dir.2026.263812 41742713

[61] Tejani AS Klontzas ME Gatti AA Mongan JT Moy L Park SH. Checklist for artificial intelligence in medical imaging (CLAIM): 2024 update. Radiol Artif Intell. (2024) 6(4):e240300. 10.1148/ryai.240300 38809149 PMC11304031

[62] Vasey B Nagendran M Campbell B Clifton DA Collins GS Denaxas S. Reporting guideline for the early-stage clinical evaluation of decision support systems driven by artificial intelligence: DECIDE-AI. Nat Med. (2022) 28(5):924–33. 10.1038/s41591-022-01772-9 35585198

[63] Muneer A Zhang K Hamdi I Qureshi R Waqas M Fouad S. Foundation models in biomedical imaging: turning hype into reality. Nat Biomed Eng. (2026) 10:1557–75. 10.1038/s41551-026-01762-z 42595819

[64] Mahdi MA Al-Shalabi M Elbarougy R Alnfrawy ET Hadi MU Ali RF. CT-to-PET synthesis in the head–neck and thoracic region via conditional 3D latent diffusion modeling. Bioengineering. (2026) 13(5):534. 10.3390/bioengineering13050534 42194291 PMC13203646

[65] Brady AP Allen B Chong J Kotter E Kottler N Mongan J. Developing, purchasing, implementing and monitoring AI tools in radiology: practical considerations. A multi-society statement from the ACR, CAR, ESR, RANZCR & RSNA. Can Assoc Radiol J. (2024) 75(2):226–44. 10.1177/08465371231222229 38251882

[66] U.S. Food and Drug Administration. Artificial Intelligence-Enabled Device Software Functions: Lifecycle Management and Marketing Submission Recommendations. U.S. Food and Drug Administration (2025).

[67] U.S. Food and Drug Administration. Marketing Submission Recommendations for a Predetermined Change Control Plan for Artificial Intelligence-Enabled Device Software Functions. U.S. Food and Drug Administration (2025).

